# Immunization of Mice with Gold Nanoparticles Conjugated to Thermostable Cancer Antigens Prevents the Development of Xenografted Tumors

**DOI:** 10.3390/ijms232214313

**Published:** 2022-11-18

**Authors:** Lev A. Dykman, Sergey A. Staroverov, Sergey V. Kozlov, Alexander S. Fomin, Daniil S. Chumakov, Konstantin P. Gabalov, Yevgeny S. Kozlov, Dmitry A. Soldatov, Nikolai G. Khlebtsov

**Affiliations:** 1 Institute of Biochemistry and Physiology of Plants and Microorganisms, Saratov Scientific Centre of the Russian Academy of Sciences, Saratov 410049, Russia; 2 Veterinary Faculty, Vavilov Saratov State Agrarian University, Saratov 410012, Russia; 3 Institute of Physics, Saratov State University, Saratov 410012, Russia

**Keywords:** gold nanoparticles, heat shock proteins, vaccination, adjuvant, tumor transplantation

## Abstract

Gold nanoparticles as part of vaccines greatly increase antigen stability, antigen accumulation in the lymph nodes, and antigen uptake by antigen-presenting cells. The use of such particles as part of anticancer vaccines based on heat shock proteins to increase vaccine effectiveness is timely. We prepared and characterized nanoconjugates based on 15-nm gold nanoparticles and thermostable tumor antigens isolated from MH22a murine hepatoma cells. The whole-cell lysate of MH22a cells contained the main heat shock proteins. BALB/c mice were injected with the conjugates and then received transplants of MH22a cells. The highest titer was produced in mice immunized with the complex of gold nanoparticles + antigen with complete Freund’s adjuvant. The immunized mice showed no signs of tumor growth for 24 days. They also showed a decreased production of the INF-γ, IL-6, and IL-1 proinflammatory cytokines compared to the mice immunized through other schemes. This study is the first to show that it is possible in principle to use gold nanoparticles in combination with thermostable tumor antigens for antitumor vaccination. Antitumor vaccines based on thermostable tumor antigens can be largely improved by including gold nanoparticles as additional adjuvants.

## 1. Introduction

The immunotherapy of malignant tumors is an important biomedical research area. In time, it may take its place alongside surgery, chemotherapy, and radiotherapy as a standard in cancer treatment. Current cancer immunotherapies include cytokine therapy [1], immune checkpoint inhibition [2], adaptive cell therapy [3], and antitumor vaccines [4]. Anticancer vaccines stimulate antitumor immunity. The basic idea behind such vaccines is that malignant cells overexpress tumor antigens to which a T-cell immune response can be mounted. Any antitumor vaccine includes the following elements: (1) an antigen, (2) a carrier, which determines the delivery of the antigen to the lymphoid organs, and (3) an adjuvant, which enhances the immunogenicity of the antigen [5]. Whether this approach is successfully transferred from laboratory to clinic depends critically on the overcoming of the associated problems. First, the administered vaccine may accumulate poorly in lymph nodes. Second, the antigen may be inefficiently processed and presented by dendritic cells. This prevents the induction of a sufficient CD8^+^ T-cell response [6].

Nanoscale materials can help to solve these problems. Organic and inorganic nanoparticles in tumor vaccines greatly increase the stability of an antigen and its accumulation in lymph nodes, uptake, processing, and cross-presentation by antigen-presenting cells [7,8,9]. Among all available nanoparticles for this purpose, one of the most popular is gold nanoparticles (GNPs) [10]. They act both as antigen carriers and as adjuvants [11]. GNPs can serve as adjuvants to improve the effectiveness of vaccines, stimulate antigen-presenting cells, and provide a controlled release of antigens. GNPs are chemically inert, biocompatible, and easy to make. In addition, their surface can easily be functionalized with biomolecules, including antigens of various natures [12,13]. The unique plasmon resonance properties of GNPs make it possible to track the biodistribution of GNP-based vaccines and develop multimodal nanocomposites, which combine efficient antigen delivery with photothermal activity and imaging possibilities [14]. GNPs are popularly used to design antibacterial, antiviral, and antiparasitic vaccines [15,16].

The work of Brinãs et al. [17] was one of the first to report on the induction in laboratory animals of an immune response to tumor antigens conjugated to colloidal gold without the use of additional adjuvants. The authors used GNPs conjugated to a glycopeptide antigen based on the MUC4 mucin, a modified Thomsen–Friedenreich antigen, and a peptide from the complement-derived protein C3d. Twelve weeks after the vaccination, there was a statistically significant increase in the blood content of IgG and IgM antibodies. Parry et al. [18] reported the preparation of antibodies specific to the tumor monosaccharide Tn-antigen without the use of conventional protein components of vaccines. Animals were immunized with GNPs conjugated to a polymerized Tn-antigen. Cai et al. [19] showed that animals immunized with PEGylated GNPs conjugated to a glycopeptide sequence derived from MUC1 and the T-cell epitope P30 sequence developed Th1 and Th2 immune responses. The resultant antiserum contained antibodies that recognized MUC-1 molecules on the surface of MCF-7 breast cancer cells. In vitro, GNPs complexed with MUC-1 mucin increased the production of the TNF-ɑ, IL-6, IL-10, and IL-12 cytokines by peritoneal macrophages and induced macrophage polarization [20]. In addition, GNPs coupled to the adjuvant of ɑ-galactosylceramide were used to enhance the immune response to MUC-1 [21].

Almeida et al. [22] experimentally validated a GNP-based peptide antitumor nanovaccine. GNPs coated with the ovalbumin epitope were injected subcutaneously into mice. The animals injected with the conjugate showed a significant increase in INF-γ production by splenocytes. Seven days after the last immunization, the animals were challenged with B16-OVA melanoma cells. The immunized animals survived 100% within 50 days of the experiment. Ahn et al. [23] studied the response of RAW 264.47 macrophages and of dendritic and T cells isolated from mice to GNPs conjugated to peptides derived from tumor cell antigens. The GNP–peptide complexes induced 15-fold higher TNF-ɑ production by macrophages than did the free antigen. The complexes also enhanced antigen cross-presentation by dendritic cells, which was manifested as an increased elaboration of IFN-γ and IL-2 by T-lymphocytes.

The size and associated surface curvature, shape, surface charge, and hydrophobicity of the surface are some of the key characteristics that affect the consequences of nanoparticle uptake on immune cells [24]. The small size of nanoadjuvants gives them special properties that other adjuvants do not have. Due to the small size of the nanoparticles, they can penetrate the lymphatic organs better, and can also be used as a carrier to load immune adjuvants. Small nanoadjuvants more easily pass through biological barriers and are more effective in realizing the stability of blood circulation [25]. Smaller-sized GNPs allow for greater tissue distribution, deeper tissue penetration, and increased cellular internalization [26].

GNPs are also used as a carrier in the development of antitumor DNA vaccines. Specifically, Gulla et al. [27] used 24-nm gold nanospheres conjugated to the DNA sequence encoding the MART1 melanoma antigen (pCMV–MART1) to immunize mice and examine their response to subsequent tumor cell transplantation. Additionally, the authors coated the nanoconjugates with a mannose-mimicking shikimoyl ligand to target dendritic cells and a guanidine ligand to facilitate transfection. The nanovaccine-immunized mice survived the entire experimental period (180 days) without signs of tumor after subcutaneous melanoma transplantation. Cytotoxic lymphocyte depletion studies showed that CD8^+^ T-lymphocytes are the key link in the antitumor immune response when the nanoconjugates are injected. Of further note are works in which GNPs were used as siRNA carriers to inhibit immune response checkpoints [28,29]. Thus, GNPs are promising candidates for improving the efficacy and safety of cancer immunotherapy [30].

Heat shock proteins (HSPs) can play an important part in the induction of an antitumor adaptive immune response [31]. HSPs are primarily known as highly conserved chaperone proteins involved in the folding, assembly, and disassembly of protein complexes [32]. Their chaperone activity underlies the mediation of efficient capture, processing, and cross-presentation of tumor peptide antigens by dendritic cells [33]. On the other hand, HSPs can also modulate the innate immune response by participating in NK cell activation [34]. They promote the production of chemokines and pro- and anti-inflammatory cytokines such as IL-1, IL-6, IL-10, IL-12, TNF-α, and IFN-γ [35,36]. Thermostable proteins of the HSP family have been popularly used as adjuvants in the design of vaccines against a variety of cancers [37,38,39]. Some of these developments have reached phase III trials [40]. Phase I and II trials of therapeutic vaccines containing HSP–peptide complex-96 (HSPPC-96) for glioblastoma treatment are underway [41].

In the use of HSP-based vaccines, the immune response can be enhanced with nanoscale materials. Shevtsov et al. [42] coupled iron oxide nanoparticles to recombinant HSP 70 for orthotopic experimental glioblastoma therapy in animals. Seven days after tumor cell transplantation, the animals were immunized with a mixture of dendritic cells, tumor antigens derived from the lysate, and nanoconjugates. The animals in the experimental group showed significant inhibition of tumor growth and the activation of a CD8^+^ T-cell response, as compared to the group immunized with a nanoparticle-free dendritic cell–HSP 70–antigen mixture.

Despite enormous publications on the application of GNPs in cancer research, there are no related reports on the use of GNPs in combination with HSPs for antitumor vaccination. Here, we investigated the part that GNPs play in the induction of antitumor immunity in laboratory mice. We immunized BALB/c mice with conjugates of 15-nm GNPs to thermostable tumor antigens isolated from MH22a murine hepatoma cells. Then, the immunized mice received transplants of MH22a cells. By contrast, to control animals, the immunized mice demonstrated completely inhibited tumor growth for 24 days and a decreased production of the INF-γ, IL-6, and IL-1 proinflammatory cytokines. To the best of our knowledge, this is the first demonstration of using gold nanoparticles in combination with thermostable tumor antigens for antitumor vaccination. 

## 2. Results

### 2.1. Characterization of GNPs

**Figure 1:**
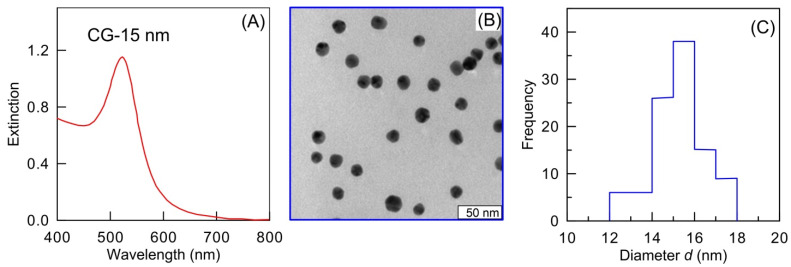
Characterization of GNPs: extinction spectrum (**A**), TEM image (**B**), and size distribution (DLS data) (**C**).

The extinction spectrum peak of fabricated GNPs is located at 518 nm, and the extinction in a 1-cm cuvette is close to 1.2 (Figure 1A). These plasmonic parameters are typical for 15-nm colloids with a gold concentration of 0.3 mM. As found by TEM, the mean nanoparticle diameter was 15.2 ± 1.2 nm (Figure 1B). The number concentration of particles was 1.9 × 10^12^ particles/mL. Our earlier work showed the citrate-stabilized quasi-spherical GNPs with a mean diameter of 15 nm to be optimal for use in immunization [43].

### 2.2. Characterization of Antigens

**Figure 2:**
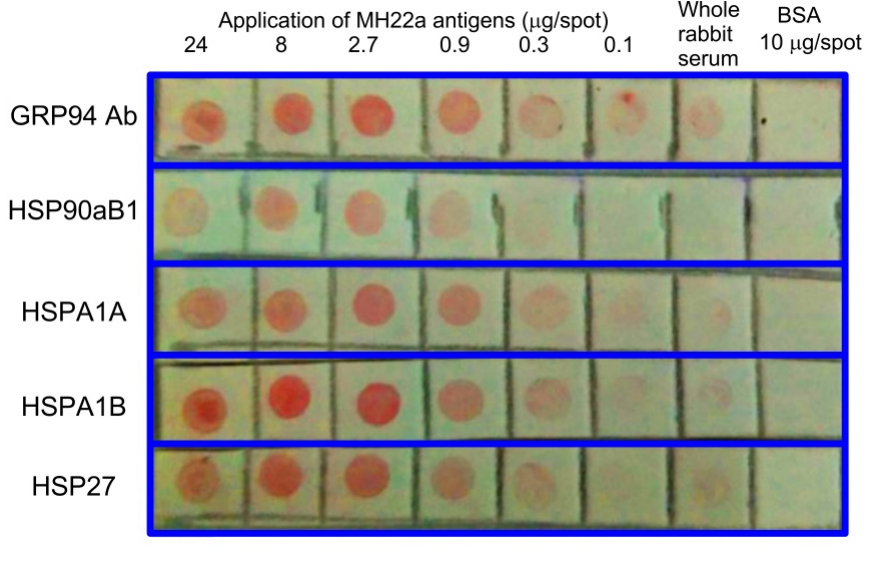
Identification of HSPs in the whole-cell lysate of MH22a by dot blot immunoassay.

Figure 2 shows the results for the content of the main HSPs in the whole-cell lysate of MH22a cells. The results were obtained by dot blot immunoassay using a set of rabbit anti-HSP polyclonal antibodies. As can be seen, the lysate of heated MH22a cells contained all main HSPs, including GRP94, HSP 90 kDa alpha B1 (HSP90aB1), HSP 70 kDa 1A (HSPA1A), HSP 70 kDa 1B (HSPA1B), and HSP 27 kDa.

### 2.3. Immunization Results

Table 1 shows the obtained antibody titers. The highest titer (1:10,666 on average; maximum titer, 1:12,800) was produced in mice immunized with the GNP–antigen–CFA complex. Immunization with the antigen and GNPs separately gave average titers of 1:1200 (maximal titer, 1:1600) and 1:1066 (maximal titer, 1:1600), respectively. The average titer in mice immunized with the GNP–antigen conjugate was 1:366 (maximal titer, 1:800). When mice were immunized with GNPs alone, the presence of an antibody titer and the ability of antibodies to bind to the cellular antigen were probably related to the immune response of the animals to the tumor cell transplantation. In the blot assay of the MH22a HSPs, the resulting antiserum specifically recognized peptides ranging in size from 25 to 66 kDa.

**Figure 3:**
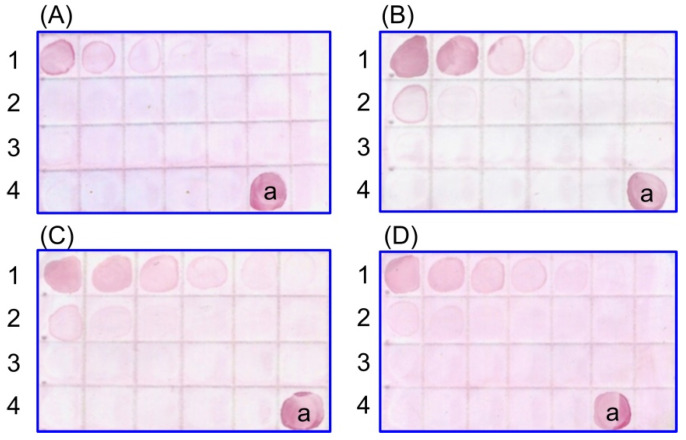
Dot immunoassay of antigens with antisera from mice immunized with antigen (**A**), GNPs + antigen (**B**), GNPs + antigen + CFA (**C**), and GNPs (**D**). 1, MN22a. 2, HeLa. 3, SPEV-2. 4a, serum (positive control).

The specificity of the antisera obtained from the immunized mice was examined by dot immunoassay (Figure 3). Note that the sera interacted mainly with the MN22a cell antigens. However, the sera obtained from the mice immunized with the GNP–antigen–CFA complex and those immunized with GNPs alone gave a small crossover with the HeLa cell antigens.

**Figure 4:**
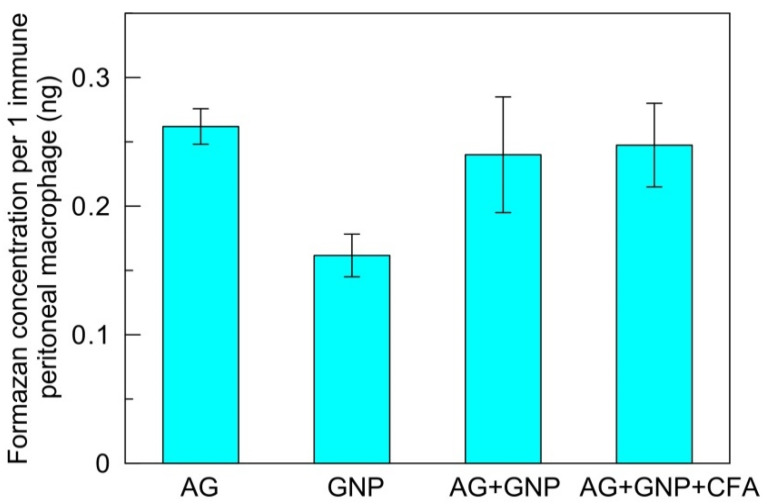
Changes in the respiratory activity of peritoneal macrophages in mice immunized by different schemes. The differences between the mean values for AG, AG + GNP, and AG + GNP + CFA variants are not statistically significant.

Figure 4 shows the results for the respiratory activity of peritoneal macrophages isolated from animals of different groups after immunization and subsequent tumor transplantation. As found by the MTT test, there were no statistically significant differences between the groups of animals injected with the antigen alone, the GNP–antigen conjugate, and the GNP–antigen–CFA complex. However, in the animals injected with GNPs alone, the respiratory activity of peritoneal macrophages decreased slightly.

**Figure 5:**
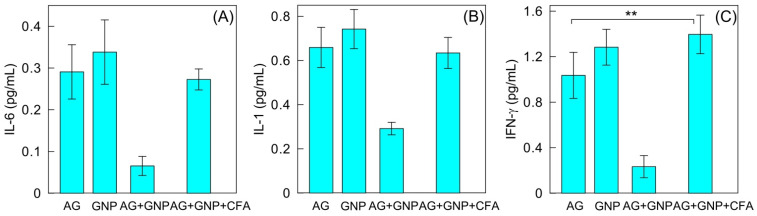
Content of IL-6 (**А**), IL-1I (**B**), and FN-γ (**C**) in the sera of mice after immunization and subsequent tumor transplantation. The difference between the mean values for AG and AG + GNP + CFA variants is statistically significant in panel (**C**). ** means significant difference.

Figure 5 shows the results for the content of proinflammatory cytokines in the sera of mice after immunization and subsequent tumor transplantation. In the mice immunized with the GNP–antigen conjugate, the production of proinflammatory cytokines was significantly reduced, as compared to that in the other groups. IFN-γ production decreased by an average of 79%; IL-6 production, by an average of 80%; and IL-1 production, by an average of 57%. In the other groups of mice, no significant differences were noted between the amounts of proinflammatory cytokines produced. An exception presents a statistically significant difference between AG and AG + GNP + CFA variants.

### 2.4. Tumor Formation Results

**Figure 6:**
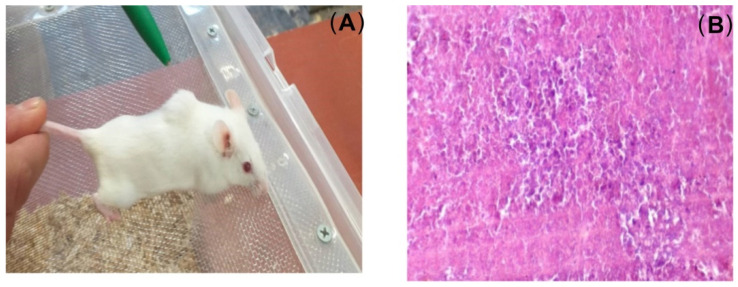
The appearance of a mouse with a transplanted tumor on day 24 after transplantation (Tumor size 2.5 cm) (**A**), photograph by S. Staroverov. Histological section of the tumor on day 24 after transplantation (**B**). Staining with hematoxylin–eosin, ×300.

**Figure 7:**
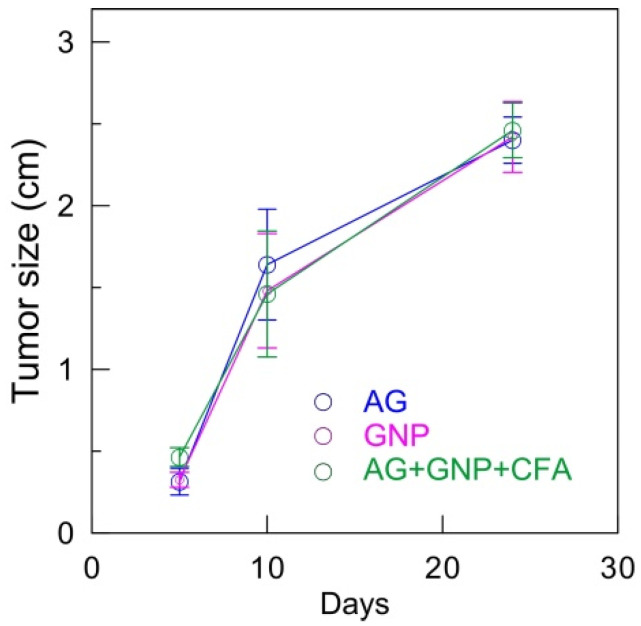
Tumor growth dynamics after immunization and subsequent tumor cell transplantation.

Seven days after the last immunization, the mice received transplants of MH22a tumor cells. In groups 1, 3, and 4, variously sized tumors (0.5–2.5 cm; Figure 6A) were present in all animals. On histological examination of the tumors, no hepatic lobule structure, characteristic of the liver parenchyma, was observed. Nested cell clusters were well visualized. The tumor cells were morphologically similar to hepatocytes but were larger than them; they were polygonal, with globular nuclei, and clearly defined karyosomes. Between neighboring tumor cells, there were ring-shaped structures characteristic of hepatomas (Figure 6B), yet, no tumors were observed at the injection site in any group 2 mouse (GNPs + Ag). Only one mouse had a small lump on day 21, but the lump disappeared on day 24. In groups 1, 3, and 4, the transplanted tumor cells survived 100%. Tumors appeared on day 5. Tumor sizes were measured on days 5, 10, and 24 (Figure 7).

**Figure 8:**
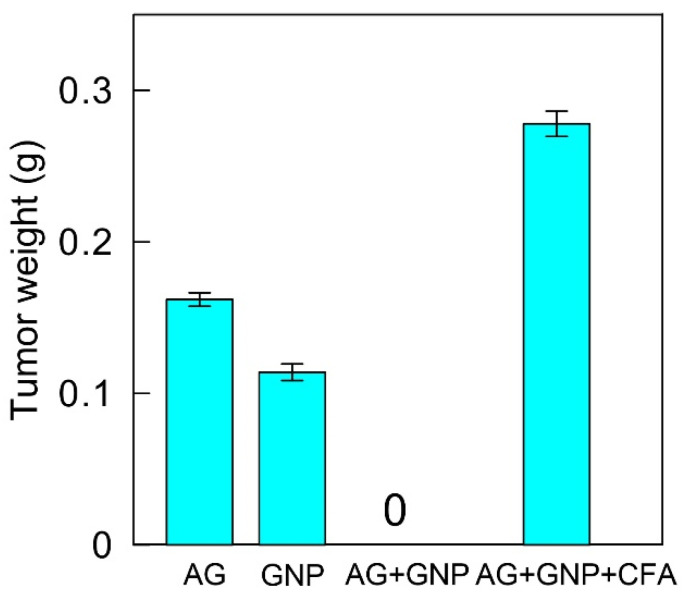
Tumor weight after immunization, subsequent tumor cell transplantation, and euthanasia after 24 days.

When the growth dynamics were constant, the tumor size in the infected mice was maximal on day 24, but tumor weight varied depending on the immunization method. Specifically, tumor weight was the largest in the animals immunized with the GNP–antigen–CFA complex—0.287 g, which is actually twice as large as the values obtained for the animals immunized with the antigen and GNPs alone (Figure 7 and Figure 8).

## 3. Discussion

**Figure 9:**
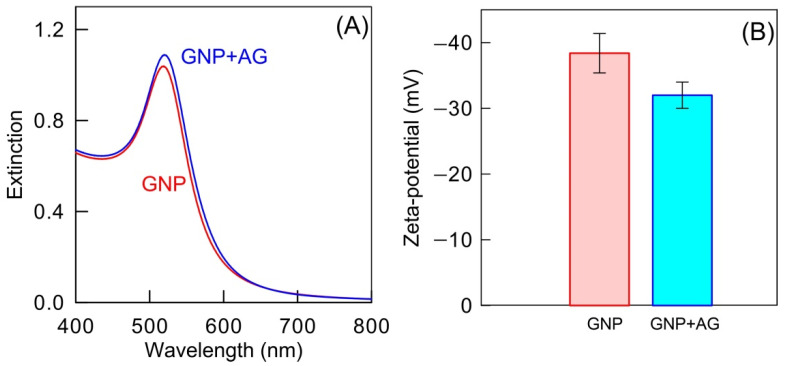
Extinction spectra (**A**) and zeta-potentials (**B**) of GNPs before (**red**) and after (**blue**) conjugation with AG.

The stability of conjugated gold nanoparticles is a crucial parameter for their application as carriers of specific molecules. Typically, the particle aggregation results in a notable broadening of the plasmonic peak and its shifting to longer wavelengths. Figure 9A demonstrates the absence of any spectral changes after the conjugation of citrate-stabilized as-prepared particles with AG proteins. Additional confirmation of the conjugate stability comes from zeta potential measurements (Figure 9B). At pH = 7.5, the zeta potential of as-prepared gold nanoparticles has a typical value between −30 and −40 mV (−38.4 mV in our case), thus indicating the electrostatic nature of colloidal stability. After conjugation with AG proteins, zeta potential is decreased slightly in absolute value, remaining negative (−32.5 mV). Therefore, the stability of conjugated particles can be explained by two contributions: electrostatic repulsion and steric factors.

HSPs play an important role in tumor processes as they are involved in various cancer-related activities such as cell proliferation, metastasis, and resistance to anticancer drugs [44]. HSP functions are associated with the onset, progression, and metastasis of cancer, as well as resistance to cancer therapy. In addition, the potential use of HSPs to enhance the effects of chemo-, radio-, and immunotherapy is being studied. HSPs have many clinical applications as biomarkers for cancer diagnosis and prognosis, and as potential therapeutic targets for anticancer treatments [45]. The use of HSPs in antitumor vaccines is very promising, using their ability to act as immunological adjuvants.

Analysis of the results shows that animal immunization with the GNP–antigen conjugate prevents tumor formation after cancer cell transplantation. This is even though the antibody titer produced by conjugate immunization was the lowest. This may be because in this case, immunization produces a Th1-dependent immune response. It is accompanied by the elaboration of IFN-γ, IL-2, and TNF-α, which affect the production of opsonizing and complement-binding antibodies by B-cells, the activation of macrophages, cytotoxicity, and the induction of cellular immunity. Immune responses dominated by Th1 cells mostly cause phagocyte-dependent inflammation [46,47]. The number of T cells, especially activated CD8^+^ cytotoxic T cells and Th1 cells, correlates with better survival in some cancers, including invasive colorectal cancer, melanoma, multiple myeloma, and pancreatic cancer [48].

In the mice injected with the GNP–antigen–CFA complex, tumor induction could have been due to an inflammatory process in the tumor microenvironment that could have been caused by immunization with CFA and by increased contents of IL-1β, IL-6, IL-8, and monocyte chemoattractant protein-1 (MCP-1), observed in breast cancer patients [49,50]. It has been suggested that these mediators can directly promote the proliferation and invasion of breast cancer cells or can participate in angiogenesis, which is important for breast cancer development and progression [51].

Kitamura et al. [52] reported that IL-6 suppresses the major histocompatibility complex (MHC) class II expression on Th1 cells, inhibiting IFN-γ and IL-2 secretion. They noted that in this case, the cancer cells evaded antitumor immunological effects through the reduction in cytotoxic T lymphocyte activity. This has also been pointed out in studies on the relationship between IL-6 and gastrointestinal and other cancers, and a role was found for IL-6 in the development and maintenance of neoplastic cells. Gastric cancer cells secrete IL-6, and increased serum and tumor-tissue amounts of IL-6 possibly regulate tumor growth and development. Also, increased amounts of inflammatory mediators such as TNF-α, C-reactive protein, and IL-6 have been found in the sera of hepatocellular carcinoma patients [53,54].

## 4. Materials and Methods

### 4.1. Preparation of GNPs

Gold nanospheres were synthesized as described by Frens [55], by reducing HAuCl_4_ with sodium citrate. A 240 mL portion of deionized water was heated to boiling in an Erlenmeyer flask fitted with a water-cooled reflux tube. This was followed by the addition to the flask of 2.5 mL of 1% aqueous HAuCl_4_ (Sigma–Aldrich, St. Louis, MO, USA) and 7.75 mL of 1% sodium citrate (Fluka, Buchs, Switzerland). The mixture was vigorously stirred. The mean particle size was examined by spectrophotometry, transmission electron microscopy (TEM, Libra 120, Carl Zeiss, Oberkochen, Germany), and dynamic light scattering (DLS, Zetasizer Nano ZS, Malvern Instruments, Malvern, UK).

### 4.2. Culturing of Cells

MH22a murine hepatoma cells, HeLa cervical carcinoma cells, and SPEV-2 porcine embryonic kidney cells were used. All cells were obtained from the Russian Collection of Cell Cultures of the Russian Academy of Sciences’ Institute of Cytology, St. Petersburg, Russia. Cells were grown on Dulbecco’s modified Eagle’s medium (DMEM) with 10% fetal bovine serum, 100 units/mL penicillin, 100 μg/mL streptomycin, and 292 μg/mL L-glutamine. They were grown in sterile adhesive-culture bottles until monolayers were formed.

### 4.3. Isolation of the Total HSP Fraction

After monolayers were formed, HSPs were isolated as described previously [56,57]. The culture bottle with the MH22a monolayer was heated at 42 °C for 1 h and incubated at 37 °C for 2 h. The cells were lysed, and the bottle was gently washed four times with 10 mL of heated Hanks solution. Then, 15 mL of DMEM containing 4 mM glutamine and 2 mM phenylmethylsulfonyl fluoride (PMSF) was added. After the bottle was shaken at 37 °C for 1 h and was washed, it received 10 mL of Cytomix buffer (120 mM KCl, 0.15 mM CaCl_2_, 2 mM EDTA, 5 mM MgCl_2_, 10 mM K_2_HPO_4_/KH_2_PO_4_, 25 mM HEPES, and 2 mM PMSF, pH 8.0) and, finally, was frozen at −20 °C and thawed at 37 °C. This procedure was repeated three times. The suspension was then transferred to a centrifuge tube and spun at 10,000× *g* for 15 min. Alternatively, cells were removed from the monolayer by trypsin treatment and 7 × 10^6^ tumor cells were lysed as described above. The resultant samples were clarified by centrifugation at 20,000× *g* for 20 min. Protein was measured by the Bradford method and was frozen at −70 °C.

The cell lysate was then precipitated with ammonium sulfate to 40% saturation, and the precipitate was spun at 20,000× *g* for 20 min at 4 °C. The resulting supernatant liquid was precipitated with ammonium sulfate to a final saturation of 80%, and the precipitate was spun at 20,000× *g* for 20 min at 4 °C. The sediment obtained after the second centrifugation was dissolved in 4 mL distilled water and dialyzed against 0.2 M phosphate-buffered saline (PBS), pH 7.2, at 4 °C for 48 h with frequent buffer changes. Antigens from HeLa and SPEV-2 cells were isolated in the same manner. The resultant extracts were used for further chromatographic purification.

### 4.4. Chromatographic Purification of Antigens

Antigens were purified by ion-exchange chromatography on a Toyopearl DEAE-650 column (Sigma–Aldrich, St. Louis, MO, USA), using an NGC Quest 10 chromatograph (Bio-Rad, Hercules, CA, USA). The phase was equilibrated with 0.05 M Tris–HCl, pH 7.5. The equilibrated sample (100 μL), containing 240 μg protein, was applied to the column. The eluates were collected as fractions by using a stepwise gradient of 0 to 0.5 M NaCl. The absorbance of the eluates was monitored at 280 nm with a Spectronic-21 spectrophotometer (Thermo Scientific, Waltham, MA, USA).

### 4.5. Preparation of Conjugates

A 150-µL portion of staphylococcal protein A (concentration, 1 mg/mL) and 25 mL of colloidal gold solution (absorbance *A*_520_ = 1) were mixed. The mixture was stirred for 10 min. Then, 500 μL of 1% PEG-20,000 was added to the reaction mixture and the mixture was stirred for another 5 min. The samples were centrifuged at 12,000× *g* for 40 min and the supernatant liquid was then decanted. The sediment was redissolved in a buffer composed of 10 mM PBS, 0.02 M NaN_3_, 0.02% PEG-20,000, and 30% glycerol so that the absorbance *A*_518_ of the sample was 5.

Before conjugation, we estimated the “gold number” (minimal amount of antigen that protects the sol against salt aggregation) of the GNPs with thermostable antigens derived from the MH22a whole-cell lysate. To this end, 20 μL of an aqueous antigen solution (initial concentration, 1 mg/mL) was titrated twofold on a 96-well microtiter plate. Each well received 200 μL of 15-nm GNPs (absorbance *A*_520_ = 1.0) and 20 μL of 1.7 M NaCl. The minimal stabilizing concentration for the isolated antigen was 12 μg/mL. Conjugation was done by simple mixing, and no coupling agents were used. The antigen concentration used exceeded the gold number by 20%. Of note, an excess of a soluble antigen not only does not interfere with immunization but facilitates an increase in antibody production [58]. The conjugate remained stable during the three-month follow-up.

### 4.6. Characterization of GNPs and Conjugates

Extinction spectra of GNPs and GNP+AG were recorded with a Specord S-300 spectrophotometer (Analytik Jena, Jena, Germany). Transmission electron microscopy images were obtained with a Libra-120 transmission electron microscope (Carl Zeiss, Oberkochen, Germany). The zeta potential of particles and conjugates was measured using the dynamic light scattering method with a Zetasizer Nano ZS, Malvern Instruments, Malvern, UK). All measurements were carried out at the Simbioz Center for the Collective Use of Research Equipment in the Field of Physico-Chemical Biology and Nanobiotechnology, IBPPM RAS, Saratov.

### 4.7. Dot Blot Immunoassay

The dot immunoassay was run as follows [59]: Extracts from MH22a cells were applied as a series of spots onto a Western S polyvinylidene fluoride membrane (Millipore, Burlington, MA, USA). The membrane was blocked for 1 h with 2% fat-free powdered milk diluted in 10 mM PBS, pH 7.2, and was then incubated for 1 h in a solution of antibodies prediluted 1:150. The isolated HSPs were identified by using mouse polyclonal antibodies against GRP94 (Affinity Bioscience, Cincinnati, OH, Germany), HSP 90 kDa alpha B1 (HSP90aB1), HSP 70 kDa 1A (HSPA1A), HSP 70 kDa 1B (HSPA1B), and HSP 27 kDa (Cloud-Clone, Katy, TX, USA). When there was a biospecific interaction, the antibodies bound to the antigen adsorbed on the membrane. The membrane was then washed free of nonspecifically bound antibodies and was immersed in a solution of GNPs conjugated to staphylococcal protein A (absorbance *А*_520_ = 1). After 5–60 min, the binding of the conjugate to the antigen-antibody complex was observable visually as a series of red spots.

### 4.8. Western Blotting

First, sodium dodecyl sulfate-polyacrylamide gel electrophoresis (SDS–PAGE) was carried out according to Laemmli [60]. Molecular weight marker protein standards (Sigma–Aldrich, St. Louis, MO, USA) were included in each gel. After electrophoresis, the gels were stained with Coomassie Brilliant Blue R-250 (Sigma–Aldrich, St. Louis, MO, USA). For Western blotting, the electrophoresed samples were transferred to a Western S membrane with a semidry blotter and the membrane was incubated for 1 h in a blocking buffer containing 10 mM PBS, 0.1% Tween 20, and 5% fat-free powdered milk. Finally, the membrane was incubated for 1 h in an antibody-containing serum solution and the reaction results were detected with GNPs conjugated to staphylococcal protein A (absorbance *А*_518_ = 1).

### 4.9. Examination of Antitumor Efficacy of GNP–Antigen Conjugates

GNPs complexed with thermostable antigens derived from the MH22a whole-cell lysate were used to immunize BALB/c white mice (18–20 g). The mice were acquired from the Department of Experimental Animals with Vivarium, Russian Research Anti-Plague Institute “Microbe”, Saratov, Russian Federation. The animals were divided into six groups of five each. Group 1 received a PBS solution of the antigen (3 μg; 250 μL); group 2, the GNP–antigen conjugate (3 μg; 250 μL); group 3, the conjugate emulsified 1:1 with complete Freund’s adjuvant (CFA) (3 μg; 500 μL); and group 4, a solution of GNPs (250 μL). The animals were immunized intraperitoneally by two injections with an interval of 10 days in between. Seven days after the last immunization, the animals received subcutaneous transplants of MH22a tumor cells in the area of the back. These were injected into the withers at 1 × 10^9^ cells/mouse. The first signs of tumors appeared 14 days after infection; on day 21, the tumors were most clearly visible. On day 24, the animals were killed and blood was drawn to determine the antibody titer and the interleukin content. In addition, peritoneal cells were isolated to measure respiratory activity (MTT test) and tumor histology was evaluated.

Animals were cared for and handled under the Guide for the Care and Use of Laboratory Animals, the European Convention for the Protection of Vertebrate Animals Used for Experimental and Other Scientific Purposes, and the legislation of the Russian Federation. The use of animals was also approved by the institution where the experiments were performed.

### 4.10. Enzyme-Linked Immunosorbent Assay

The antibody titer was estimated by enzyme-linked immunosorbent assay (ELISA) with horseradish peroxidase-labeled secondary antibodies against mouse IgG (Jackson Immuno Research, Cambridge, UK) [61]. The reaction results were recorded on a plate screen analyzer (Hospitex Diagnostics, Sesto Fiorentino, Italy). Animal sera were diluted 10-fold and then doubly titrated. The serum interleukin concentrations were measured by ELISA with reagent kits for IL-1β, IL-6, and IFN-γ (Vector-Best, Novosibirsk, Russia).

### 4.11. Examination of Cellular Respiratory Activity

For isolating peritoneal macrophages, the animals were killed and then fixed on their backs. An incision was made along the midline of the anterior abdominal wall and the skin flap was carefully separated, with care taken to keep the peritoneum intact. After a puncture had been made with a needle connected to a syringe, 50 mL of PBS, pH 7.2, was injected into the peritoneal cavity. The anterior abdominal wall was then gently massaged, and after 5–7 min, peritoneal fluid was collected with a Pasteur pipet through a cut made in the peritoneum and was filtered into a test tube through a nylon filter. The cells were washed three times by centrifugation in PBS at 750× *g*, after which they were resuspended in 1 mL of PBS and counted in a Goryaev chamber. Peritoneal macrophages were cultured using standard procedures [62].

The respiratory activity of peritoneal macrophages was measured by the ability of the cells to reduce nitrotetrazolium blue [3-(4,5-dimethylthiazol-2-yl)-2,5-diphenyltetrazolium bromide] to formazan (MTT test) [63]. Experimental and control cells (200 μL of each kind) were added to the wells of a microtitration plate and the plate was placed in a CO_2_ incubator. After 72 h, 20 μL of 0.5% MTT solution was added to all wells and the plate was incubated for another 3.5 h. An MTT stock solution (5 mg/mL) was made with PBS and stored at 4 °C in a dark vessel for no more than 2 weeks. A 165–170-μL portion of the supernatant liquid was then carefully taken from the wells and 150 μL of DMSO was added to dissolve the formed formazan crystals. The contents of the wells were carefully pipetted; alternatively, a micro shaker was used to shake the plates. The absorbance of the solution was measured on a Spark-10M microplate reader (Tecan, Männedorf, Switzerland) at 560 nm. The proliferation coefficient was calculated from the formula *К* = *A*_exp_/*A*_control_.

### 4.12. Pathomorphological Studies

The animals were killed by cervical dislocation under anesthesia. Tumor size was measured with a micrometer with a division value of 0.1 mm. For histological studies, whole tumors were placed in a container with a 10% neutral aqueous buffered solution of formaldehyde, 96° alcohol, and Carnoy’s fixative. From the fixed tumors, paraffin blocks (Histomix embedding medium; BioVitrum, Saint-Petersburg, Russia) were made by standard procedures. Sections were сut on a MICROM HM 450 sliding microtome (Germany).

For microscopic observation, sections were stained with hematoxylin–eosin by the Ehrlich method. The stained sections were embedded into Canadian balm (Panreac, Barcelona, Spain) under a coverslip and were examined with a Micromed S-1 biological microscope (Biomed, Saint-Petersburg, Russia). Microphotographs were taken with a CANON PowerShot A460 IS camera (Canon, Tokyo, Japan).

### 4.13. Statistics

The results were statistically processed using the standard procedures integrated into Excel 2007 software (Microsoft, Redmond, WA, USA). After the arithmetic mean value and the standard deviation for a given data sample had been found, the standard error of the arithmetic mean and its confidence limits were determined, taking into account the Student’s *t* coefficient (*n*, *p*) [number of measurements *n* = 3, significance level = 95% (*p* = 0.05)]. These results are presented as histograms. The significance of differences between individual samples was evaluated using a two-sample unpaired Student’s *t*-test with unequal variances. Differences were considered significant when the experimentally found *p*_exp_ value was ≤0.05.

## 5. Conclusions

Recent years have seen the intense development of effective adjuvants for antitumor immunotherapy [64]. In particular, HSPs [65,66] and GNPs of various shapes and sizes have been proposed [67,68,69]. Here, we used both of these adjuvants to examine the part they play in the induction of antitumor immunity in laboratory mice.

We prepared conjugates based on 15-nm GNPs and thermostable antigens isolated from MH22a murine hepatoma cells. BALB/c mice were immunized by different schemes and then received transplants of MH22a cells. The mice immunized with the GNP–antigen conjugate showed no signs of tumor growth for 24 days. None of the mice in this group developed a tumor, whereas mice in all the other groups did. The mice immunized with the nanoconjugate had the lowest antibody titer. They also showed a significantly decreased production of the INF-γ, IL-6, and IL-1 proinflammatory cytokines.

Our main conclusion is the possibility, at least in principle, to use GNPs in combination with HSPs for antitumor vaccination. The results suggest that anticancer vaccines can be largely improved by including GNPs as additional adjuvants. In future work, we plan to investigate cytotoxic T cells activity and the level of different molecular markers, such as PD-1, TIM-3, LAG-3, NKG2A, and CTLA-4, in CD8+ T cells of immunized and unimmunized mice. The next stage of research could be to design and test therapeutic vaccines based on HSPs and colloidal gold.

## Figures and Tables

**Table 1:** Antibody titers produced by different immunization schemes after tumor cell transplantation.

Immunogen	Antibody Titer			Student’s *t*-Test Relative to the Antigen (*p* ≤ 0.05)
	Average Titer	Maximal Titer	Average Titer (log_2_)	
Аntigen	1:1200	1:1600	10.14 ± 0.9	
GNPs + antigen	1:366	1:800	8.14 ± 2.03	0.092
GNPs + antigen + CFA	1:10,666	1:12,800	13.31 ± 0.8	0.00062
GNPs	1:1066	1:1600	9.997 ± 0.8	0.074

## Data Availability

The data underlying this article will be shared on reasonable request to the corresponding author.

## References

[1] Waldmann T.A. (2018). Cytokines in cancer immunotherapy. Cold Spring Harb. Perspect. Biol..

[2] Robert C. (2020). A decade of immune-checkpoint inhibitors in cancer therapy. Nat. Commun..

[3] Rohaan M.W., Wilgenhof S., Haanen J.B.A.G. (2019). Adoptive cellular therapies: The current landscape. Virchows Arch..

[4] Apostolopoulos V. (2019). Cancer vaccines: Research and applications. Cancers.

[5] Finn O.J. (2003). Cancer vaccines: Between the idea and the reality. Nat. Rev. Immunol..

[6] Lou J., Zhang L., Zheng G. (2019). Advancing cancer immunotherapies with nanotechnology. Adv. Ther..

[7] Wang X., Li X., Ito A., Watanabe Y., Sogo Y., Tsuji N.M., Ohno T. (2016). Stimulation of in vivo antitumor immunity with hollow mesoporous silica nanospheres. Angew. Chem..

[8] Luo M., Wang H., Wang Z., Cai H., Lu Z., Li Y., Du M., Huang G., Wang C., Chen X. (2017). A STING-activating nanovaccine for cancer immunotherapy. Nat. Nanotechnol..

[9] Zhou L., Hou B., Wang D., Sun F., Song R., Shao Q., Wang H., Yu H., Li Y. (2020). Engineering polymeric prodrug nanoplatform for vaccination immunotherapy of cancer. Nano Lett..

[10] Salazar-González J.A., González-Ortega O., Rosales-Mendoza S. (2015). Gold nanoparticles and vaccine development. Expert Rev. Vaccines.

[11] Dykman L.A., Khlebtsov N.G. (2017). Immunological properties of gold nanoparticles. Chem. Sci..

[12] Alkilany A.M., Murphy C.J. (2010). Toxicity and cellular uptake of gold nanoparticles: What we have learned so far?. J. Nanopart. Res..

[13] Dykman L.A., Khlebtsov N.G. (2019). Methods for chemical synthesis of colloidal gold. Rus. Chem. Rev..

[14] Dreaden E.C., Alkilany A.M., Huang X., Murphy C.J., El-Sayed M.A. (2012). The golden age: Gold nanoparticles for biomedicine. Chem. Soc. Rev..

[15] Dykman L.A. (2020). Gold nanoparticles for preparation of antibodies and vaccines against infectious diseases. Expert Rev. Vaccines.

[16] Burygin G.L., Abronina P.I., Podvalnyy N.M., Staroverov S.A., Kononov L.O., Dykman L.A. (2020). Preparation and in vivo evaluation of glyco-gold nanoparticles carrying synthetic mycobacterial hexaarabinofuranoside. Beilstein J. Nanotechnol..

[17] Brinãs R.P., Sundgren A., Sahoo P., Morey S., Rittenhouse-Olson K., Wilding G.E., Deng W., Barchi J.J. (2012). Design and synthesis of multifunctional gold nanoparticles bearing tumor-associated glycopeptide antigens as potential cancer vaccines. Bioconjug. Chem..

[18] Parry A.L., Clemson N.A., Ellis J., Bernhard S.S., Davis B.G., Cameron N.R. (2013). ‘Multicopy multivalent’ glycopolymer-stabilized gold nanoparticles as potential synthetic cancer vaccines. J. Am. Chem. Soc..

[19] Cai H., Degliangeli F., Palitzsch B., Gerlitzki B., Kunz H., Schmitt E., Fiammengo R., Westerlind U. (2016). Glycopeptide-functionalized gold nanoparticles for antibody induction against the tumor associated mucin-1 glycoprotein. Bioorg. Med. Chem..

[20] Mocan T., Matea C., Tabaran F., Iancu C., Orasan R., Mocan L. (2015). In vitro administration of gold nanoparticles functionalized with MUC-1 protein fragment generates anticancer vaccine response via macrophage activation and polarization mechanism. J. Cancer.

[21] Liu Y., Wang Z., Yu F., Li M., Zhu H., Wang K., Meng M., Zhao W. (2021). The adjuvant of α-galactosylceramide presented by gold nanoparticles enhances antitumor immune responses of MUC1 antigen-based tumor vaccines. Int. J. Nanomed..

[22] Almeida J.P.M., Lin A.Y., Figueroa E.R., Foster A.E., Drezek R.A. (2015). In vivo gold nanoparticle delivery of peptide vaccine induces anti-tumor immune response in prophylactic and therapeutic tumor models. Small.

[23] Ahn S., Lee I.H., Kang S., Kim D., Choi M., Saw P.E., Shin E.C., Jon S. (2014). Gold nanoparticles displaying tumor-associated self-antigens as a potential vaccine for cancer immunotherapy. Adv. Healthc. Mater..

[24] Sharma P., Alakesh A., Jhunjhunwala S. (2022). The consequences of particle uptake on immune cells. Trends Pharmacol. Sci..

[25] Tan J., Ding B., Teng B., Ma P., Lin J. (2022). Understanding structure–function relationships of nanoadjuvants for enhanced cancer vaccine efficacy. Adv. Funct. Mater..

[26] Chen X.-Y., Yung L.-Y.L., Tan P.H., Bay B.H. (2022). Harnessing the immunogenic potential of gold nanoparticle-based platforms as a therapeutic strategy in breast cancer immunotherapy: A mini review. Front. Immunol..

[27] Gulla S.K., Rao B.R., Moku G., Jinka S., Nimmu N.V., Khalid S., Patra C.R., Chaudhuri A. (2019). In vivo targeting of DNA vaccines to dendritic cells using functionalized gold nanoparticles. Biomater. Sci..

[28] Gulla S.K., Kotcherlakota R., Nimushakavi S., Nimmu N.V., Khalid S., Patra C.R., Chaudhuri A. (2018). Au-CGKRK nanoconjugates for combating cancer through T-cell-driven therapeutic RNA interference. ACS Omega.

[29] Zhang Y., Fu J., Shi Y., Peng S., Cai Y., Zhan X., Song N., Liu Y., Wang Z., Yu Y. (2018). A new cancer immunotherapy via simultaneous DC-mobilization and DC-targeted IDO gene silencing using an immune-stimulatory nanosystem. Int. J. Cancer.

[30] He J.-S., Liu S.-J., Zhang Y.-R., Chu X.-D., Lin Z.-B., Zhao Z., Qiu S.-H., Guo Y.-G., Ding H., Pan Y.-L. (2021). The application of and strategy for gold nanoparticles in cancer immunotherapy. Front. Pharmacol..

[31] Shevtsov M., Multhoff G. (2016). Heat shock protein-peptide and HSP-Based immunotherapies for the treatment of cancer. Front. Immunol..

[32] Schlesinger M.J. (1990). Heat shock proteins. J. Biol. Chem..

[33] Murshid A., Gong J., Stevenson M.A., Calderwood S.K. (2011). Heat shock proteins and cancer vaccines: Developments in the past decade and chaperoning in the decade to come. Expert Rev. Vaccines.

[34] Multhoff G., Pfister K., Gehrmann M., Hantschel M., Gross C., Hafner M., Hiddemann W. (2001). A 14-mer Hsp70 peptide stimulates natural killer (NK) cell activity. Cell Stress Chaperones.

[35] Basu S., Srivastava P.K. (2000). Heat shock proteins: The fountainhead of innate and adaptive immune responses. Cell Stress Chaperones.

[36] Tsan M.F., Gao B. (2004). Cytokine function of heat shock proteins. Am. J. Physiol. Cell Physiol..

[37] Mazzaferro V., Coppa J., Carrabba M.G., Rivoltini L., Schiavo M., Regalia E., Mariani L., Camerini T., Marchianò A., Andreola S. (2003). Vaccination with autologous tumor-derived heat-shock protein gp96 after liver resection for metastatic colorectal cancer. Clin. Cancer Res..

[38] Maki R.G., Livingston P.O., Lewis J.J., Janetzki S., Klimstra D., Desantis D., Srivastava P.K., Brennan M.F. (2007). A phase I pilot study of autologous heat shock protein vaccine HSPPC-96 in patients with resected pancreatic adenocarcinoma. Dig. Dis. Sci..

[39] Pilla L., Patuzzo R., Rivoltini L., Maio M., Pennacchioli E., Lamaj E., Maurichi A., Massarut S., Marchianò A., Santantonio C. (2006). A phase II trial of vaccination with autologous, tumor-derived heat-shock protein peptide complexes Gp96, in combination with GM-CSF and interferon-alpha in metastatic melanoma patients. Cancer Immunol. Immunother..

[40] Testori A., Richards J., Whitman E., Mann G.B., Lutzky J., Camacho L., Parmiani G., Tosti G., Kirkwood J.M., Hoos A. (2008). Phase III comparison of vitespen, an autologous tumor-derived heat shock protein gp96 peptide complex vaccine, with physician’s choice of treatment for stage IV melanoma: The C-100-21 Study Group. J. Clin. Oncol..

[41] Ampie L., Choy W., Lamano J.B., Fakurnejad S., Bloch O., Parsa A.T. (2015). Heat shock protein vaccines against glioblastoma: From bench to bedside. J. Neurooncol..

[42] Shevtsov M.A., Nikolaev B.P., Yakovleva L.Y., Parr M.A., Marchenko Y.Y., Eliseev I., Yudenko A., Dobrodumov A.V., Zlobina O., Zhakhov A. (2016). 70-kDa heat shock protein coated magnetic nanocarriers as a nanovaccine for induction of anti-tumor immune response in experimental glioma. J. Control. Release.

[43] Dykman L.A., Staroverov S.A., Fomin A.S., Khanadeev V.A., Khlebtsov B.N., Bogatyrev V.A. (2018). Gold nanoparticles as an adjuvant: Influence of size, shape, and technique of combination with CpG on antibody production. Int. Immunopharmacol..

[44] Albakova Z., Siam M.K.S., Sacitharan P.K., Ziganshin R.H., Ryazantsev D.Y., Sapozhnikov A.M. (2021). Extracellular heat shock proteins and cancer: New perspectives. Trans Oncol..

[45] Staroverov S.A., Kozlov S.V., Brovko F.A., Fursova K.K., Shardin V.V., Fomin A.S., Gabalov K.P., Soldatov D.A., Zhnichkova E.G., Dykman L.A. (2022). Phage antibodies against heat shock proteins as tools for in vitro cancer diagnosis. Biosens. Bioelectron. X.

[46] DeNardo D.G., Barreto J.B., Andreu P., Vasquez L., Tawfik D., Kolhatkar N., Coussens L.M. (2009). CD4+ T cells regulate pulmonary metastasis of mammary carcinomas by enhancing protumor properties of macrophages. Cancer Cell.

[47] Laghi L., Bianchi P., Miranda E., Balladore E., Pacetti V., Grizzi F., Allavena P., Torri V., Repici A., Santoro A. (2009). CD3+ cells at the invasive margin of deeply invading (pT3-T4) colorectal cancer and risk of post-surgical metastasis: A longitudinal study. Lancet Oncol..

[48] Grivennikov S.I., Greten F.R., Karin M. (2010). Immunity, inflammation, and cancer. Cell.

[49] Goldberg J.E., Schwertfeger K.L. (2010). Proinflammatory cytokines in breast cancer: Mechanisms of action and potential targets for therapeutics. Curr. Drug Targets.

[50] Gilbert C.A., Slingerland J.M. (2013). Cytokines, obesity, and cancer: New insights on mechanisms linking obesity to cancer risk and progression. Annu. Rev. Med..

[51] Nicolini A., Carpi A., Rossi G. (2006). Cytokines in breast cancer. Cytokine Growth Factor Rev..

[52] Kitamura H., Ohno Y., Toyoshima Y., Ohtake J., Homma S., Kawamura H., Takahashi N., Taketomi A. (2017). Interleukin-6/STAT3 signaling as a promising target to improve the efficacy of cancer immunotherapy. Cancer Sci..

[53] Vainer N., Dehlendorff C., Johansen J.S. (2018). Systematic literature review of IL-6 as a biomarker or treatment target in patients with gastric, bile duct, pancreatic and colorectal cancer. Oncotarget.

[54] Wang H., Liu J., Hu X., Liu S., He B. (2016). Prognostic and therapeutic values of tumor necrosis factor-alpha in hepatocellular carcinoma. Med. Sci. Monit..

[55] Frens G. (1973). Controlled nucleation for the regulation of the particle size in monodisperse gold suspensions. Nat. Phys. Sci..

[56] Aguilera R., Saffie C., Tittarelli A., Gonzalez F.E., Ramírez M., Reyes D., Pereda C., Hevia D., García T., Salazar L. (2011). Heat-shock induction of tumor-derived danger signals mediates rapid monocyte differentiation into clinically effective dendritic cells. Clin. Cancer Res..

[57] Skarga Y., Vrublevskaya V., Evdokimovskaya Y., Morenkov O. (2009). Purification of the 90 kDa heatshock protein (hsp90) and simultaneous purification of hsp70/hsc70, hsp90 and hsp96 from mammalian tissues and cells using thiophilic interaction chromatography. Biomed. Chromatogr..

[58] Tao W., Gill H.S. (2015). M2e-immobilized gold nanoparticles as influenza A vaccine: Role of soluble M2e and longevity of protection. Vaccine.

[59] Dykman L.A., Bogatyrev V.A. (1997). Colloidal gold in solid-phase assays. A review. Biochemistry.

[60] Laemmli U.K. (1970). Cleavage of structural proteins during the assembly of the head of bacteriophage T4. Nature.

[61] Shah K., Maghsoudlou P. (2016). Enzyme-linked immunosorbent assay (ELISA): The basics. Br. J. Hosp. Med..

[62] Leiter E.H. (1997). The NOD mouse: A model for insulin dependent diabetes mellitus. Curr. Protoc. Immunol..

[63] Bernas T., Dobrucki J.W. (2000). The role of plasma membrane in bioreduction of two tetrazolium salts, MTT, and CTC. Arch. Biochem. Biophys..

[64] Maeng H.M., Berzofsky J.A. (2019). Strategies for developing and optimizing cancer vaccines. F1000Research.

[65] Lei Y., Zhao F., Shao J., Li Y., Li S., Chang H., Zhang Y. (2019). Application of built-in adjuvants for epitope-based vaccines. Peer J..

[66] Banstola A., Jeong J.-H., Yook S. (2020). Immunoadjuvants for cancer immunotherapy: A review of recent developments. Acta Biomater..

[67] Chauhan A., Khan T., Omri A. (2021). Design and encapsulation of immunomodulators onto gold nanoparticles in cancer immunotherapy. Int. J. Mol. Sci..

[68] Zhou L., Liu H., Liu K., Wei S. (2021). Gold compounds and the anticancer immune response. Front. Pharmacol..

[69] Toraskar S., Chaudhary P.M., Kikkeri R. (2022). The shape of nanostructures encodes immunomodulation of carbohydrate antigen and vaccine development. ACS Chem. Biol..

